# Population genetics of *Glossina palpalis palpalis *from central African sleeping sickness foci

**DOI:** 10.1186/1756-3305-4-140

**Published:** 2011-07-18

**Authors:** Trésor Tito Tanekou/TT Melachio, Gustave Simo, Sophie Ravel, Thierry De Meeûs, Sandrine Causse, Philippe Solano, Pascal Lutumba, Tazoacha Asonganyi, Flobert Njiokou

**Affiliations:** 1 Université de Yaoundé I, Laboratoire de Parasitologie et Ecologie, Faculté des Sciences, BP 812, Yaoundé, Cameroun; 2 Department of Biochemistry, Faculty of Science, University of Dschang, PO Box 67, Dschang, Cameroon; 3 Institut de Recherche pour le Développement (IRD), UMR IRD-CIRAD INTERTRYP, Campus international de Baillarguet, 34398 Montpellier cedex 05, France; 4 Centre International de Recherche-Développement sur l'Elevage en zone Subhumide (CIRDES), 01 BP 454, Bobo-Dioulasso 01, Burkina-Faso; 5 CNRS, Délégation Languedoc-Roussillon, 1919, route de Mende - 34293 Montpellier cedex 5, France; 6 Department of Parasitology, University of Kinshasa, Democratic Republic of Congo; 7 Faculty of Medicine and Biomedical Sciences, University of Yaoundé 1, Yaounde-Cameroon

## Abstract

**Background:**

*Glossina palpalis palpalis *(Diptera: Glossinidae) is widespread in west Africa, and is the main vector of sleeping sickness in Cameroon as well as in the Bas Congo Province of the Democratic Republic of Congo. However, little is known on the structure of its populations. We investigated *G. p. palpalis *population genetic structure in five sleeping sickness foci (four in Cameroon, one in Democratic Republic of Congo) using eight microsatellite DNA markers.

**Results:**

A strong isolation by distance explains most of the population structure observed in our sampling sites of Cameroon and DRC. The populations here are composed of panmictic subpopulations occupying fairly wide zones with a very strong isolation by distance. Effective population sizes are probably between 20 and 300 individuals and if we assume densities between 120 and 2000 individuals per km^2^, dispersal distance between reproducing adults and their parents extends between 60 and 300 meters.

**Conclusions:**

This first investigation of population genetic structure of *G. p. palpalis *in Central Africa has evidenced random mating subpopulations over fairly large areas and is thus at variance with that found in West African populations of *G. p. palpalis*. This study brings new information on the isolation by distance at a macrogeographic scale which in turn brings useful information on how to organise regional tsetse control. Future investigations should be directed at temporal sampling to have more accurate measures of demographic parameters in order to help vector control decision.

## Background

Human African Trypanosomiasis (HAT) is a neglected tropical disease occurring in sub-Saharan Africa. After several historical cycles of epidemics followed by decreases in prevalence [1], WHO has recently announced the aim of elimination of HAT as a public health problem [2]. Central Africa, in particular DRC, remains the most affected area by sleeping sickness, harbouring more than 90% of the total number of cases [3].

The distribution of HAT foci depends on the combined presence of the parasite, the vertebrate host, and the tsetse. The species *Glossina palpalis*, which is the main vector of HAT in West Africa, and which is also a vector of HAT in Central Africa, and a vector of animal trypanosomiasis in western and central Africa, is composed of two subspecies, *G. p. gambiensis *and *G. p. palpalis*. Although several studies on tsetse population genetics have been published on *G. p. gambiensis *[4], very few data are available on the population genetic structure of *G. p. palpalis *in central Africa [5,6]. Investigations on population genetic structure of *G. p. gambiensis *have allowed observervations on genetic structuring at microgeographical scales and have allowed to measure genetic isolation between populations, which has in turn allowed control programmes to choose their control strategy (i.e. eradication or suppression) [7-9].

In the present work, we undertook a population genetic analysis of *G. p. palpalis *coming from different sleeping sickness foci of Cameroon and DRC using microsatellite DNA markers.

## Methods

### Study sites

This study was undertaken in four HAT foci of Cameroon (Bafia, Bipindi, Campo and Fontem) and one HAT focus (Malanga) of the Democratic Republic of Congo. Each Cameroon focus is separated from the other by at least 100 km. No vector control activity has been undertaken in these foci so far.

**Figure 1:**
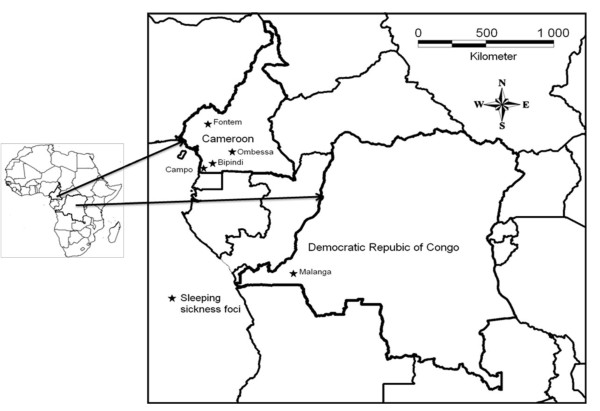
**Map showing the geographic location of samples in Cameroon and DRC**.

- Bipindi and Campo are in the South Region of Cameroon (see map in Figure 1). In both foci, previous studies identified the presence of four different tsetse species, among which *G. p. palpalis *was the most caught [10,11]. Bipindi (3°2'N, 10°22'E) is an old HAT focus known since 1920 and covers several villages that are mainly located along roads [12]. The vegetation is an equatorial forest interspersed by farmland located along the roads. This region is surrounded by hills and has a dense hydrographic network with fast running streams. It remains the most active focus of Cameroon with about 70 patients detected between 1999 and 2006. Campo (2°20'N, 9°52'E) is a hypo-endemic focus where no epidemic outbreak has been reported for several years [13]. This focus lies along the Atlantic coast and extends along the Ntem River which constitutes the Cameroon and Equatorial Guinean border. It is an equatorial rain forest zone with a network of several rivers, swampy areas and marshes. Less than 35 cases were detected between 1999 and 2006.

- The Fontem sleeping sickness focus (5°40'N, 9°55'E) is in the South-West Region of Cameroon. It has been known at a HAT focus since 1949 and has a very varied topography with hills and valleys and several fast-flowing streams. The human population, domestic animals and tsetse flies are scattered in the vegetation of the valleys and hills. Previously, the Fontem focus was among the most active foci of Cameroon [14]. From 1998 to 2006, only 8 patients were detected among 16000 inhabitants examined (OCEAC, Unpublished data). *G. p. palpalis *is the only tsetse fly species known to occur in the Fontem focus [10,15].

- Bafia (4°31'N, 11°7'E) is an old HAT focus where no sleeping sickness case has been reported since 1991. It is located in the transitional zone between the forest and the savannah. *G. p. palpalis *is the main tsetse fly found in this locality. Nevertheless, other tsetse species such as *Glossina fuscipes *and *Glossina fusca *have been reported in this area.

- Malanga (4°34'S, 14°21'E) is a HAT focus located in the Bas Congo Province of the Democratic Republic of Congo. This focus lies along the Kisantu River. The vegetation is characterized by the presence of herbaceous savannah and forest relics. *G. p. palpalis *is the only *Glossina *species found in this focus.

### Entomological surveys and sampling

In Cameroon, tsetse flies were sampled in 2009 in four HAT foci, Campo in the villages (Akak, Mabiogo and Campo Beach) in March, Fontem in the villages (Bechati, Folepi and Menji) in April, Bipindi in the villages (Ebimingbang, Lambi and Memel) in July and Bafia in the village (Ombessa) in October. In each village, twelve pyramidal traps [16] were settled for six consecutive days in favourable tsetse fly biotopes. In the Democratic Republic of Congo, the entomological survey was carried out in August 2009 in the village Malanga (Kimpese) of the Bas Congo Province. In this village, twenty pyramidal traps [16] were set up for four consecutive days in tsetse fly favourable biotopes. For both Cameroon and DRC sites, tsetse flies were collected once a day. The species, sex and teneral status were identified according to routine morphological criteria [12].

From each *G. p. palpalis *individual, all legs were taken and kept into an Eppendorf tube in 95% ethanol and labelled with a code containing the trap number followed by the individual fly number. The sampling dates and the fly number were recorded in a registration book. Flies from the 12 sub-populations, except Campo Beach, Mabiogo and the two populations from DRC, came from a single trap deployed for four consecutive days. In Malanga (DRC), Campo Beach and Mabiogo, flies came from a maximum of three traps.

A total of 427 *G. palpalis palpalis *individuals was analysed. Flies were genotyped using 8 microsatellite loci: Gpg 55.3 [17], B3, B104, B110, C102 (kindly supplied by A. Robinson, Insect Pest Control Laboratory; formerly Entomology Unit, Food and Agricultural Organization of the United Nations/International Atomic Energy Agency [FAO/IAEA], Agriculture and Biotechnology Laboratories, Seibersdorf, Austria), pGp13, pGp24 [18], and GpCAG [19]. From these, B104, B110, pGp13, and 55.3 are known to be located on the X chromosome [17,18]. GpCAG and C102 have trinucleotide repeats whereas the others are dinucleotides.

### DNA extraction and analysis of microsatellite loci

In each tube containing individual tsetse fly legs, 200 μl of 5% Chelex^® ^chelating resin was added. After incubation at 56°C for 1 hour, DNA was denatured at 95°C for 30 min. The tubes were then centrifuged at 12,000 g for 2 min and frozen for later analysis.

The PCR reactions were carried out in a thermocycler (MJ Research, Cambridge, UK) as described by Ravel et al. [20] using 10 μl of the diluted supernatant from the extraction step in a final volume of 20 μl. After PCR amplification, allele bands were resolved on a 4300 DNA Analysis System from LI-COR (Lincoln, NE) after migration in 96-lane reloadable (3×) 6.5% denaturing polyacrylamide gels. This method allows multiplexing by the use of two infrared dyes (IRDye™), separated by 100 nm (700 and 800nm), and read by a two channel detection system that uses two separate lasers and detectors to eliminate errors due to fluorescence overlap. To determine the size of different alleles, a panel of 40 size markers was used. These markers have been previously generated for *G. p. gambiensis *by cloning alleles from individual tsetse flies into pGEM-T Easy Vector (Promega Corporation, Madison, WI, USA). Three clones of each allele were sequenced using the T7 primer and the Big Dye Terminator Cycle Sequencing Ready Reaction Kit (PE Applied Biosystems, Foster City, CA, USA). Sequences were analyzed on a PE Applied Biosystems 310 automatic DNA sequencer (PE Applied Biosystems) and the exact size of each cloned allele was determined. PCR products from these cloned alleles were run in the same acrylamide gel as the samples, allowing the allele size of the samples to be determined accurately. The gels were read twice by two independent readers using the LIC-OR Saga^GT ^genotyping software.

### Data analyses

Population parameters were assessed through Weir and Cockerham's unbiased estimators [21] of Wright's *F*-statistics [22] and their significance assessed through 10000 permutations with Fstat 2.9.4 ([23], updated from Goudet [24]). *F*_IS _is allele identity probability in individuals relative to allele identity between individuals from the same subsample and is thus a measure of deviation from random union of gametes within subpopulations (*F*_IS _= 0 under local panmixia). *F*_ST _is allele identity between individuals from the same subsample relative to allele identity between different subsamples and is thus a measure of genetic differentiation between subpopulations (*F*_ST _= 0 under random distribution of genotypes across subpopulations). Significance of *F*_IS _was assessed through randomizing alleles between individuals of the same subsamples and the statistics used was directly Weir and Cockerham's estimator as implemented in Fstat. Confidence intervals were computed with Jackknives over subsamples (for each locus) or bootstraps over loci (over all loci and subsamples) from Fstat output files [25].

Heterozygote deficits can be caused by Wahlund effects, null alleles, allele drop-outs or short allele dominance. Wahlund effects were first investigated through the possible role of the number of traps in a particular zone. This was investigated through a linear regression of *F*_IS _as a function of the number of traps per site. Null alleles were looked for with the software Micro-Checker v 2.2.3 [26]. For each locus, the frequency of null alleles required to explain observed deviation from the panmictic model were computed in each subsample with van Oosterhout et al.'s method [26] and Brookfield's second method [27]. These frequencies were used to compute the number of expected null homozygotes (blanks) assuming panmixia. These expected blanks numbers were summed over all subsamples for each locus and compared to the observed ones with unilateral (alternative hypothesis: there are less observed blanks than expected) exact binomial tests under R 2.1.2.0 [28]. We also regressed these number of blanks observed at each locus and over all subsamples against mean *F*_IS_'s and tested the significance of the relationship with R.

In tsetse flies, some microsatellite loci are X-linked and thus haploid in males (e.g. [29]). For these loci, data where coded as missing in males for heterozygote dependent analyses (*F*_IS_, null alleles, drop outs and short allele dominance), and homozygous for the allele present for other analyses (clustering, differentiation and linkage disequilibrium) following a routinely undertaken protocol [7,8,29,30].

To determine the relevant unit of population structure, we used the hierarchical approach implemented in the R package HierFstat 0.04-4 [31]. Four different hierarchical levels with four corresponding *F*s could be considered: The Country (Cameroon and DRC) within Total (*F*_CT_), the Village within the Country (*F*_VC_), the Site within the Village (*F*_SV_) and the Trap within the site (*F*_TS_). The significance of these different levels was tested with a *G*-based randomization test [32], the randomization unit always being lower level among the units defined by the focused level. For instance, to test the effect of the trap within site, individuals were randomized between traps of the same site. The number of randomization was set to 1000. More details on the procedures and methods implemented in HierFstat can be found elsewhere [33].

Linkage disequilibrium was assessed through the *G*-based randomization procedure per pair of locus overall subsamples, this procedure being known to be the most powerful [34]. This was implemented in Fstat with 10000 random re-associations of alleles between loci pairs. The proportion of locus pairs that were significant was compared to the expected proportion under the null hypothesis at the 5% level of significance with a unilateral exact binomial test with alternative hypothesis "there are more than 5% significant tests in the test series" (e.g. [34]). In case of significance, detection of locus pairs that were responsible for this globally significant linkage was assessed using Benjamini and Hochberg's correction method [35]. For this, the *k P*-values are ranked from the smallest to the largest, the highest *P*-value remains unchanged, the second highest *P*-value is multiplied by *k*/(*k*-1), the second by *k*(*k*-2) and so on until the smallest *P*-value that is thus multiplied by *k*. This procedure is thus as severe as the Bonferroni correction for the smallest *P*-value, and hence is very conservative (e.g. [29]) for a comment), but it is less stringent for the other *P*-values.

Sex-biased dispersal was assessed using three tests implemented in Fstat. First, Weir and Cockerham's estimate of *F*_ST_, mean (m*AI_c_*) and variance (v*AI_c_*) of Favre et al.'s corrected assignment index *AI_c _*[36] were computed separately in each sex. Next, all three statistics were submitted to a permutation procedure during which the sex of each individual is randomly re-assigned in each subsample (10,000 permutations). The observed difference between male and female *F*_ST_, the ratio of the largest to the smallest v*AI_c _*and the *AI_c_*-based *t*-statistics defined by Goudet et al. [37] were then compared to the resulting chance distributions. For the sex that has a higher dispersal rate, *F*_ST _and m*AI_c _*are expected to be smaller and v*AI_c _*is expected to be higher than for the sex that has a lower dispersal rate (see [38] for more details on these tests). This choice of statistics is motivated by the work of Goudet et al. [37] where v*AI_c _*was shown to be the most powerful statistic when migration is low (less than 10%), while *F*_ST _performs better in other circumstances. We also chose to keep m*AI_c _*because it may be more powerful in case of complex patterns of sex specific genetic structures [39,40]. Tests were all bilateral.

Isolation by distance used the two dimensional model of Rousset [41]. Under this model, the parameter *F*_ST_/(1-*F*_ST_) estimated between two subpopulations (subsamples) is a linear function of the logarithm of geographical distances Ln(*G_D_*): *F*_ST_/((1-*F*_ST_) = *b*Ln(*G_D_*)+*a *and where the slope *b *is directly a function of demographic parameters. The product of migration rate *m *by subpopulation effective size *N_e_*, i.e. the number of immigrants from neighboring sites is *Nm *= 1/(2*πb*) and the product of dispersal surface *σ*^2 ^by the effective density of individuals *D_e _*is *D_e_σ*^2 ^= 1/(4*πb*). The significance of this regression was assessed with a Mantel test of randomization of cells of one matrix [42]. All these isolation by distance procedures were undertaken with Genepop 4 [43] with 1000000 iterations for the Mantel test and georeferenced coordinates in Km of traps. The software also computes bootstrap 95% confidence intervals for the slope *b*.

Evaluating dispersal distance between adults and their parents (*σ*) requires getting a proxy for effective density of adults. For this, we used three different methods for estimating mean effective population size *N_e_*. The two first methods were linkage disequilibrium based. The first is Bartley's method [44], from Hill [45] and modified by Waples [46] and is implemented with NeEstimator [47]. The second is Waples and Do's method implemented by LDNe [48] and finally Balloux's *F*_IS _based method. This last method corresponds to heterozygote excess method from Pudovkin et al. [49] (see also [50]) corrected by Balloux [51]. It uses the fact that, in dioecious (or self incompatible) populations, alleles from females can only combine with alleles contained in males and a heterozygote excess is expected as compared to Hardy-Weinberg expectations, and this excess is proportional to the effective population size. This method was implemented using Weir and Cockerham estimator of *F*_IS _in the equation *N_e _*= 1/(-2*F*_IS_)-*F*_IS_/(1+*F*_IS_) [51] and was only applicable in subsamples and loci with heterozygote excess, thus with very few null alleles, and probably provided overestimates in our case. These mean *N_e _*were used to estimate the effective density of tsetse flies in the different sites. A very rough estimate for the surface of a site was given by the sampling surface which was of about *S *= 0.15 km^2 ^and is probably an underestimate. Hence the resulting density *D_e _*= *N_e_*/*S *is overestimated and dispersal distance *σ *= (4*πbD_e_*)^-0.5 ^represents an underestimate.

## Results

### Heterozygote deficits

Out of the 427 individuals constituting the 12 samples at the 8 microsatellite loci, mean genetic diversity (*H_s_*), observed heterozygosity (*H_o_*) and allelic richness (*R_s_*) were greater in samples from Cameroon (*H_s _*= 0.832, *H_o _*= 0.572 and *R_s _*= 11.2 respectively) than in samples from DRC (*H_s _*= 0.723, *H_o _*= 0.488 and *R_s _*= 7.1 respectively); these differences being significant (*P*-values = 0.016, 0.031 and 0.013 respectively).

**Figure 2:**
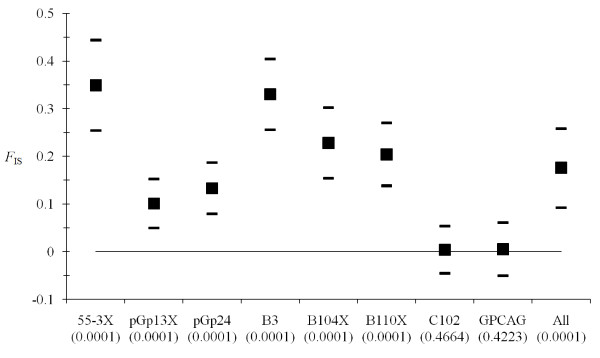
**Individual fixation index (*F*_IS_) of *G. palpalis palpalis *from Cameroon and DRC from individual traps, computed for each locus and overall (All)**. For each locus, the 95% confidence intervals were obtained by Jackknife over subsamples (individual traps) while it was obtained by bootstrap over loci for the overall mean. The *P*-value obtained while testing for significant deviation from panmixia are indicated between brackets.

**Figure 3:**
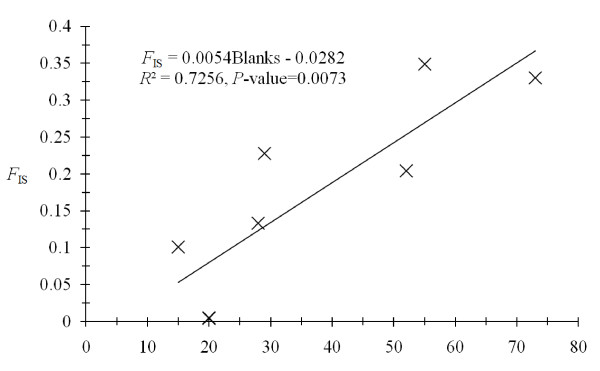
**Individual fixation index (*F*_IS_) as function of the number of blank genotypes found per locus over all sub-samples**. The equation of the regression, the determination coefficient *R*^2 ^and the significance of the *F *test (*P*-value) are also given.

Agreement with genotypic proportions expected under random mating was computed within each trap. There was an important global heterozygote deficit (*F*_IS _= 0.176, *P*-value = 0.0001). This heterozygote deficit was highly variable across loci (Figure 2). The number of available traps in a site did not explain this heterozygote deficit (*P*-value = 0.91). Using the expected number of blanks (i.e. no alleles observed) computed from Micro-Checker output, we found an agreement with the hypothesis that the observed positive *F*_IS _are explained by null alleles (minimum *P*-value > 0.433). At least 73% of the variance of *F*_IS _is explained by the number of blank genotypes found across loci (*P*-value = 0.007) (Figure 3).

### Hierarchical structure

HierFstat analysis gave a negative (hence non-significant) *F*_TS _(no trap effect) and a very small and not significant effect for sites within villages *F*_SV_~0 (*P*-value = 0.823). The only significant effect was found for villages with a *F*_VC _= 0.028 (*P*-value = 0.001), the effect of countries displaying no additional significant effect (*F*_CT _= 0.053, *P*-value = 0.092). The relevant unit that we kept for the following analyses was thus the village.

### Linkage disequilibrium

Among the 28 possible pairs of loci that could be tested, four displayed significant linkage (14%). This is more than the 5% expected under the null hypothesis (exact binomial test, *P*-value = 0.0491). None of these tests remained significant after Benjamini and Hochberg's correction. This marginally significant linkage at the genome wide scale is thus probably coming from demographic causes (small *N_e_*).

### Sex biased dispersal

Assignment based parameters were in line with a male biased dispersal but only *mAI_c _*provided a significant test (*P*-value = 0.013) (Table 1). Thus the signature of sex-biased dispersal is weak.

**Table 1:** Results of sex-biased dispersal tests on *G. palpalis palpalis *from Cameroon and DRC

	*F*_ST_	*mAI_c_*	*VarAI_c_*
Females	**0.0383**	0.2430	11.1024
Males	0.0464	**-0.5304**	**13.9234**

*P*-value	0.4931	0.0130	0.0734

Parameters used were the unbiased estimator of Wright's *F*_ST_, mean corrected assignment index (*mAI_c_*) and its variance (*varAI_c_*).

Parameters indicating the most dispersive sex are indicated in bold.

### Isolation by distance, population density and dispersal

**Figure 4:**
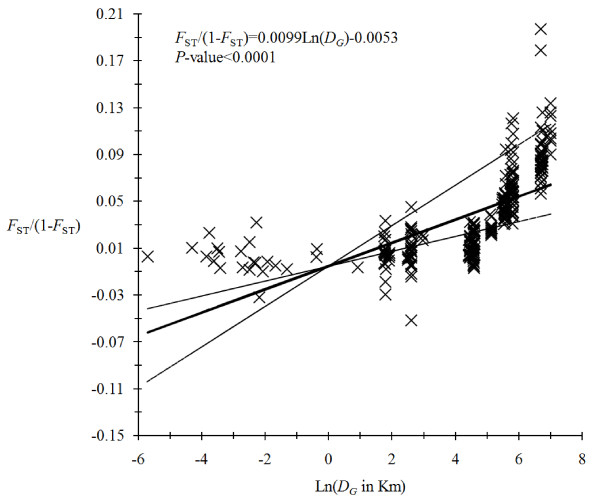
**Isolation by distance between the different *G. palpalis palpalis *captured in georeferenced traps in Cameroon and DRC**. The regression equation of Rousset's model and significance of Mantel test are indicated. The thick line is the mean model and the two thin lines correspond to those obtained from the 95% confidence intervals of the slope. More details can be found in the text.

Figure 4 shows that there is a highly significant isolation by distance with a slope *b *= 0.0099 with 95% confidence interval [0.006, 0.017], a number of migrants of *Nm *= 16 in 95% CI (9, 25) and a product *D_e_σ *^2 ^= 8 in 95% CI (5, 13).

Estimates of effective population sizes varied greatly according to the methods used (Table 2). In particular Waples and Do's method gave inconsistent values, and the reality must lie between values given by Bartley's and Balloux's methods. Hence, effective population sizes are probably between 20 and 300 individuals leading to an estimated migration rate between *m*~0.05 and *m*~0.8. Then, assuming densities stretching over between 120 and 2000 individuals per km^2^, dispersal distance between reproducing adults and their parents can be estimated between *σ*~60 and *σ*~300 meters (Table 2). The fact that Balloux's method provided several defined values of *N_e _*(in fact 17 *F*_IS _values were negative and provided an *N_e_*>0) for four loci in various subsamples (nine) is also indicative that null alleles are indeed responsible for the global heterozygote deficit observed and not a Wahlund effect.

**Table 2:** Estimation of effective population sizes with three different methods.

	LD Waples and Do	LD Bartley	*F*_IS _Balloux
*N_e_*	1442 [0, 4428]	321 [102, 539]	18 [7, 29]
*D_e _*(individuals/km^2^)	9614	2138	120
*m*	0.01 [0.006, 0.02]	0.05 [0.03, 0.08]	0.89 [0.51, 1]
*σ *(in meters)	29 [22, 36]	61 [46, 77]	258 [196, 322]

The 95% confidence intervals (*N_e _*± *t *× standard error over estimates) are indicated in square brackets, from which densities were estimated. Migration rate *m *and dispersal distance *σ *were estimated with Rousset's model of isolation by distance in two dimensions and 95% confidence intervals indicated in square brackets were computed by bootstrap over loci (see text for more details).

## Discussion

Although there have been studies dealing with *palpalis *group tsetse population structure [4,8,52], this work represents one of the first to focus on the sleeping sickness vector *G. p. palpalis *in Central Africa [see also [6]].

Population structuring was found at the geographical scale of the village, but not at the scale of the traps and above all no hidden substructure (Wahlund effect) was evidenced here, which is at variance with that reported for *G. p. palpalis *in Ivory Coast [20]. In addition, in the present work, null alleles probably explain all of the *F*_IS _that was observed. As a consequence, this high frequency of null alleles may have hampered our estimates of population structure. The effective population sizes found here (between 20 and 300 as a mean for each village) are of the same order of magnitude as found for *G. p. gambiensis *in Guinea [30]. It will be of interest to reassess these population sizes and densities in the future using temporal sampling to get more accuracy.

A slight male biased dispersal was observed, which does not correspond to typical Mark-Release-Recapture studies, these latter showing in general that females disperse more than males [53]. It is however noteworthy that using microsatellite markers, very recent studies on other tsetse species report the same trend, i.e. a male sex-biased dispersal [54,55]. Either a behaviour such as philopatry (where the females would come back to the same larviposition sites), or a sex specific local adaptation rendering immigrant females very unlikely to survive locally, may explain this.

A strong isolation by distance explains most of the population structure observed in our sampling sites of Cameroon and DRC. The populations here are composed of random mating subpopulations occupying fairly wide zones with a very strong isolation by distance that makes the probability of an allele to cross from one focus to the other (e.g: from Bipindi to Campo) very unlikely. This was reinforced by the observation of differences in number of alleles and heterozygosity between populations from Cameroon and from DRC. It has been recently reported that *G. p. palpalis *from Equatorial Guinea may constitute a different gene pool compared with *G. p. palpalis *from other countries [6]. Here, the differences in population structure as compared to Ivory-Coast counterparts might reflect again such a taxonomic heterogeneity.

Future studies on this important vector of sleeping sickness in Central Africa ought to focus on temporal sampling to better assess population densities to help targeting efficient control strategies.

## Competing interests

The authors declare that they have no competing interests.

## Authors' contributions

TDM participated to the tsetse fly sampling, the genotyping step and the manuscript drafting. GS participated to the conception, the design of the study, the tsetse fly sampling and the drifting of the manuscript. SR was involved on the genotyping step and the drifting of the manuscript. TDM performed the statistical analysis and helped to draft the manuscript. SC participated to the genotyping step and the interpretation of data. PS participated to the conception of the study, the data analysis and the drafting of the manuscript. PL participated to the conception and the tsetse fly sampling. TA participated to the conception and the design of the study. FN was involved on the conception of the study, the tsetse fly sampling and the drafting of the manuscript. All the authors read and approved the final manuscript.
